# Prior guided deep difference meta-learner for fast adaptation to stylized segmentation

**DOI:** 10.1088/2632-2153/adc970

**Published:** 2025-04-16

**Authors:** Dan Nguyen, Anjali Balagopal, Ti Bai, Michael Dohopolski, Mu-Han Lin, Steve Jiang

**Affiliations:** Medical Artificial Intelligence and Automation (MAIA) Laboratory and Department of Radiation Oncology, University of Texas Southwestern Medical Center, Dallas, TX, United States of America

**Keywords:** meta-learning, deep learning, artificial intelligence, segmentation, oncology, clinician stylization, cancer

## Abstract

Radiotherapy treatment planning requires segmenting anatomical structures in various styles, influenced by guidelines, protocols, preferences, or dose planning needs. Deep learning-based auto-segmentation models, trained on anatomical definitions, may not match local clinicians’ styles at new institutions. Adapting these models can be challenging without sufficient resources. We hypothesize that consistent differences between segmentation styles and anatomical definitions can be learned from initial patients and applied to pre-trained models for more precise segmentation. We propose a Prior-guided deep difference meta-learner (DDL) to learn and adapt these differences. We collected data from 440 patients for model development and 30 for testing. The dataset includes contours of the prostate clinical target volume (CTV), parotid, and rectum. We developed a deep learning framework that segments new images with a matching style using example styles as a prior, without model retraining. The pre-trained segmentation models were adapted to three different clinician styles for post-operative CTV for prostate, parotid gland, and rectum segmentation. We tested the model’s ability to learn unseen styles and compared its performance with transfer learning, using varying amounts of prior patient style data (0–10 patients). Performance was quantitatively evaluated using dice similarity coefficient (DSC) and Hausdorff distance. With exposure to only three patients for the model, the average DSC (%) improved from 78.6, 71.9, 63.0, 69.6, 52.2 and 46.3–84.4, 77.8, 73.0, 77.8, 70.5, 68.1, for CTV_style1_, CTV_style2_, CTV_style3_, Parotid_superficial_, Rectum_superior_, and Rectum_posterior_, respectively. The proposed Prior-guided DDL is a fast and effortless network for adapting a structure to new styles. The improved segmentation accuracy may result in reduced contour editing time, providing a more efficient and streamlined clinical workflow.

## 1. Introduction

The goal of radiotherapy is to achieve full-dose coverage of the radiation target while sparing nearby organs-at-risk (OARs). Identifying and delineating target volumes and OARs are critical for quality treatment planning and patient safety. As a standard of practice, clinicians delineate and prescribe doses to the target volume, as well as set dose-volume constraints for the OARs. Automatic segmentation for auto-contouring in radiotherapy can potentially reduce contouring time and minimize variability if it is accurate enough to not require major edits. Numerous methods for OAR segmentation have been proposed, with atlas-based methods being among the most common [1–8]. These methods align a pre-segmented atlas with the target image using affine and deformable registration. While they are robust and user-independent, atlas-based methods can still produce incorrect maps if organs are affected by tumors.

Recently, convolutional neural networks (CNNs) have significantly advanced structure delineation accuracy in radiotherapy. Ibragimov and Xing [9] introduced the first deep learning-based algorithm for OAR segmentation, using the patient’s head as a reference for OAR positions and training a patch-based CNN for voxel classification. Ren *et al* [10] developed an interleaved 3D-CNN for joint segmentation of small organs in the head and neck (H&N) region. Subsequent models by Zhu *et al* [11], Tong *et al* [12], and Tang *et al* [13] further improved H&N segmentation, and novel deep CNN architectures have also been designed for other sites [14–16]. Several papers have focused specifically on preoperative prostate gross tumor volume (GTV) and OARs [17–21], as well as on post-operative prostate clinical target volume (CTV) and OARs [22, 23]. Despite their accuracy, deploying these models in clinics poses challenges. State-of-the-art methods—such as meta-learning, domain adaptation, and transfer learning [24–28]—often require fine-tuning and significant user expertise, which can hinder clinical integration.

Common structures segmented in radiotherapy include the GTV, CTV, and OARs. A planning target volume (PTV) is then created, typically as an expansion from the CTV in order to account for additional setup error, and the final dose to deliver is prescribed to the PTV. The GTV is the palpable disease treated with radiation, while the CTV includes microscopic extensions based on guidelines and clinician experience. These variations in the CTV segmentation arise from the trade-off between OAR toxicity and tumor control, differing within and across institutions [29, 30]. Eliminating these variations is neither possible nor desirable, as the CTV definition involves judgment on many variables, with no single correct contour for a given tumor. Studies show substantial variations in contouring across different cancer types [31]. When deploying a model outside its original institution, CTV segmentation may vary due to different institutional guidelines. Additionally, OAR contours are often based on functional rather than anatomical definitions to better describe the desired spatial dose distribution. For example, in H&N cancer treatment, sparing a single lobe of the parotid gland further from the tumor can control parotid toxicity without compromising tumor coverage [32, 33]. Another similar example is the posterior/superior aspect of the rectum in prostate cancer treatment planning [34]. These contour definition variations often stem from individual clinical preferences, shifts in guidelines, or differences in clinical protocols. Consequently, the challenge of contour definition variations is a common issue and poses a significant obstacle to the deployment of segmentation models in clinics. In this paper, we collectively refer to these as style variations.

While transfer learning techniques [35–38] can adapt a pre-trained auto-segmentation model to new institutional styles, they require local clinical users to have knowledge of model adaptation and training, as well as significant hardware resources. This can be a barrier for lower volume centers. Recent studies show improved accuracy with transfer learning and test time augmentation [39, 40]. We hypothesize that the consistent differences between specific segmentation styles and anatomical definitions can be learned from the first few patients and added to the pre-trained model. This approach allows local users to correct the general model’s results for the initial patients without needing extensive data curation or model adaptation.

We introduce a Prior-guided deep difference meta-learner (Prior-guided DDL) for precise stylized segmentation. This method quickly adapts auto-segmented labels to specific styles by learning differences between a general segmentation model and specialized styles. By leveraging systematic errors from pre-trained models, our approach can distinguish segmentation style variations among clinical users for radiotherapy treatment planning. This is achieved using a pre-trained general model on several patients. To our knowledge, this is the first work addressing labeling style variability in radiotherapy treatment planning using a meta-learning approach that does not require model retraining. We tested this method on three clinical practice style variations: CTV for prostate tumor contouring, parotid gland, and rectum contouring.

## 2. Methods

### 2.1. Pipeline overview

**Figure 1.**
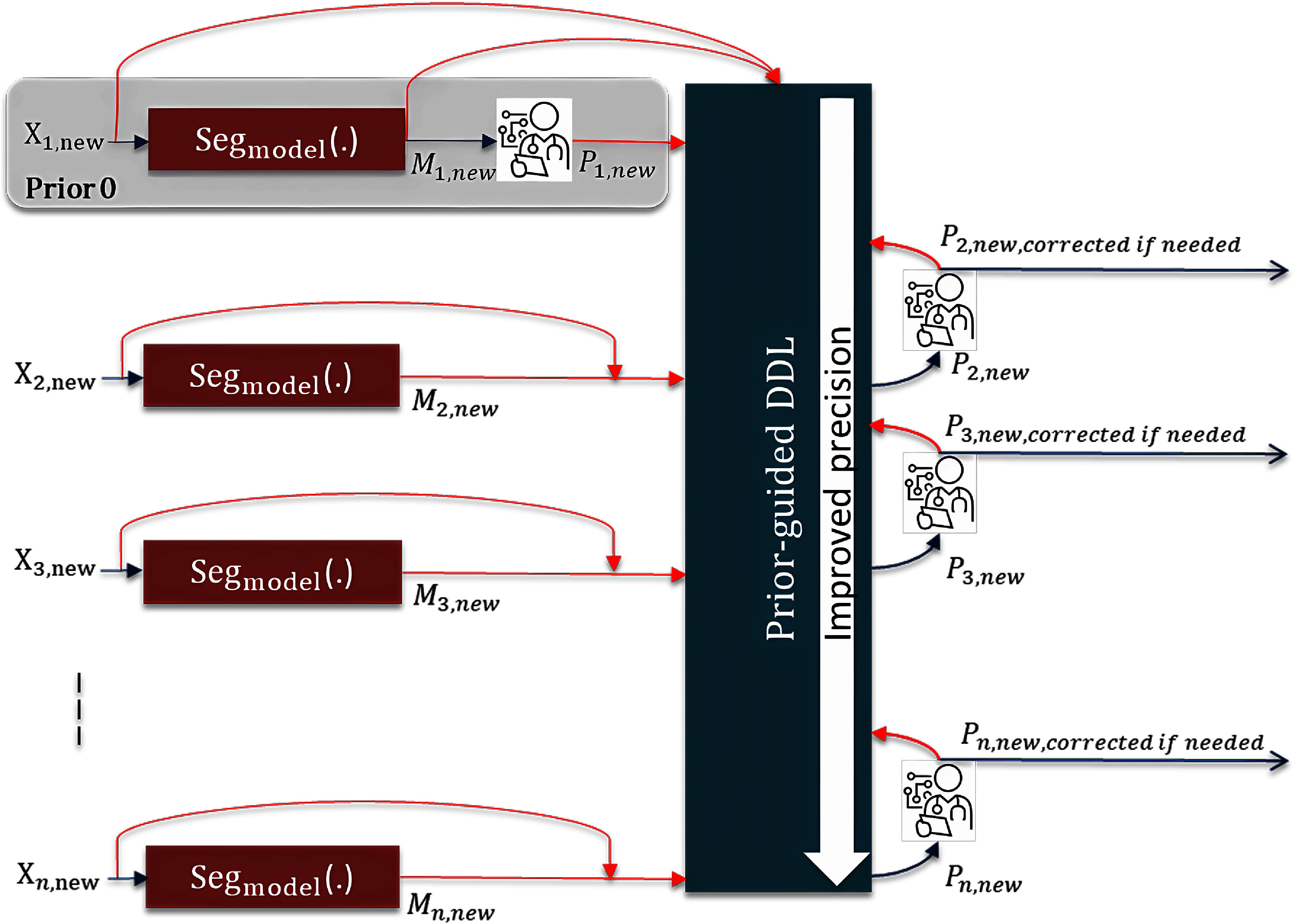
The clinical workflow for using the proposed Prior-guided DDL to a completely new segmentation style.

The clinical workflow for using the proposed Prior-guided DDL to a completely new segmentation style is illustrated in figure 1. First, a pre-trained general auto-segmentation model generates the initial contour (${M_{1,{\text{new}}}})$, and the local clinician reviews and manually corrects it to reach the new style (${P_{1,{\text{new}}}}$). This corrected patient would now serve as the first prior patient—defined in our study as a patient that has an existing segmentation, which is used to drive the segmentation model’s prediction towards a matching style for a different patient image. From the next patient onwards, the initial contour predicted by the pre-trained general auto-segmentation model is first corrected by the DDL network to generate ${P_{2,{\text{new}}}}$ with the help of ${M_{1,{\text{new}}}}{\text{ and }}{P_{1,{\text{new}}}}$, and then ${P_{2,{\text{new}}}}$ is reviewed and further corrected by the clinician if needed. The final contour ${P_{2,{\text{new}}}}$ along with ${M_{2,{\text{new}}}}{ }$ serves as the second set of prior guidance. This process is repeated with more patients. At the stage when the model acceptably adapts to the new style, the correction required by the clinician would become minimal.

### 2.2. Model architecture

**Figure 2.**
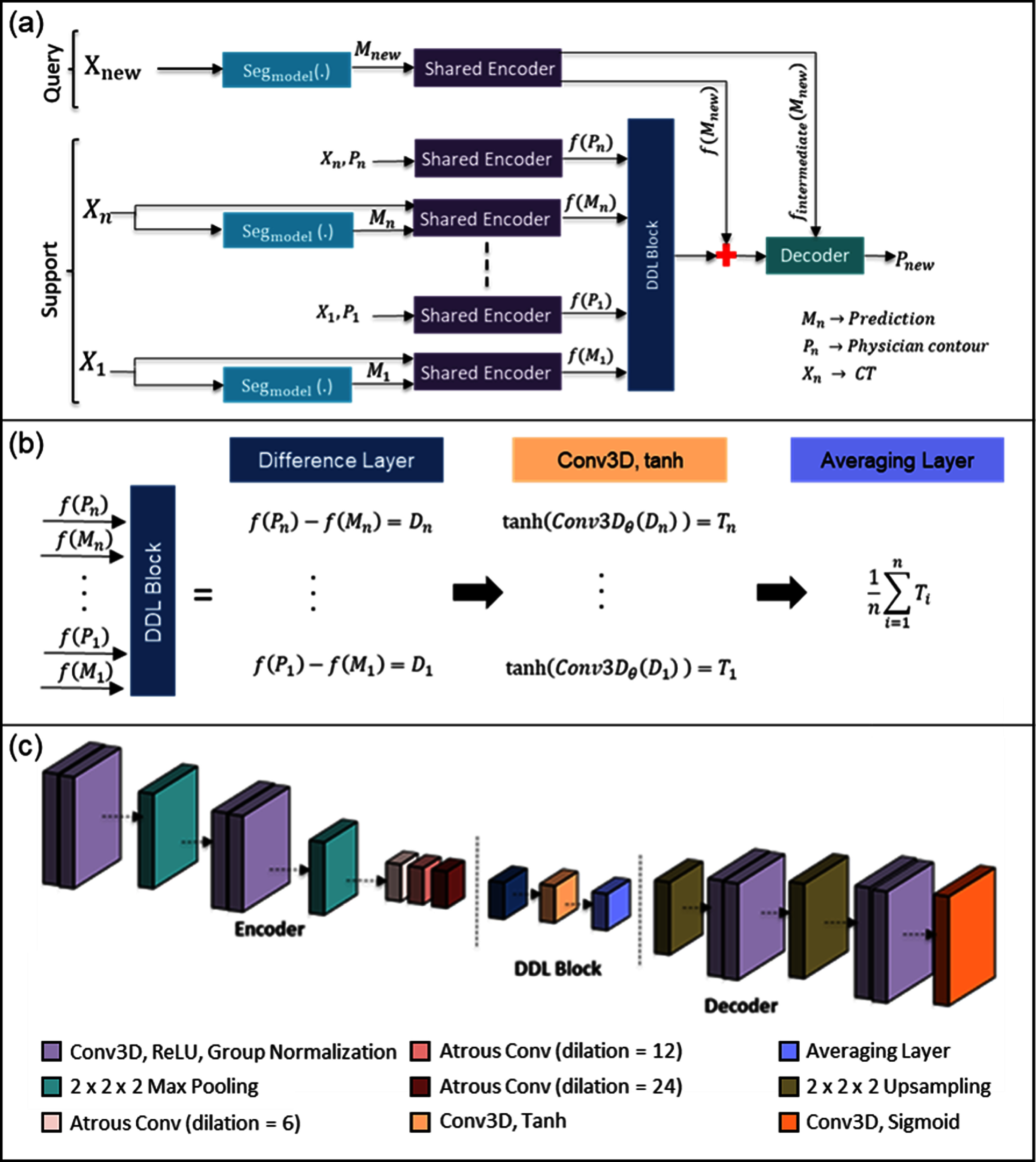
Prior-guided DDL framework. (a) Schematic of the data pipeline of the framework. (b) Detailed breakdown of the operations used in the DDL Block. (c) Architecture of the shared encoder, DDL block, and decoder. The support framework learns the style differences between the segmentation from the pre-trained auto-segmentation model and the new segmentation style. Feature differences are averaged across multiple prior patients, if available, and concatenated with the new patient features. These features are then decoded to get the style-adapted structure.

The Prior-guided DDL model has 3 goals: (1) Learn the difference between the pre-trained general segmentation model prediction and the new desired style; (2) The model should be able to predict the new style from prior patients without updating any model parameters (any further training); and (3) the model should have incremental performance with more prior patients. In this section, we first define the setup of prior guidance, and then we introduce our new Prior-guided DDL model, which learns the difference in styles and produces a stylized segmentation. Figure 2 illustrates the high-level model diagram of our method. The model has four parts. A pre-trained general segmentation model, which, from here on, will be referred to as the segmentation model, a shared encoder, a DDL block, and a decoder. The pre-trained segmentation model is the main component of prior-guidance. It generates the initial segmentation ${M_n}$ from the CT image ${X_n}$, which needs to be adapted to the new style. The style to be learned is in the form of ground truth segmentation ${P_n}$ for the same patient.

The pairs $\left\{ {{X_n},{\text{ }}{M_n}} \right\}$ and $\left\{ {{X_n},{\text{ }}{P_n}} \right\}{\text{ }}$ are input into the same encoder for feature extraction. The encoder is a multi-layer CNN. It compresses data by first processing the data in each layer, followed by a down sampling step called max pooling. Prior to max pooling, each layer processes the data via the following operations: 3D convolution (3 × 3 × 3 size kernel), ReLU activation, group normalization (32 features per group), and atrous convolution (dilation rates of 6, 12, 24).

For our implementation, the layer and max pooling step is applied twice. The encoder starts with calculating 32 features at the top resolution level and doubles the number of features after each max pooling layer, resulting in latent space data of 128 features after the atrous convolutions. The resulting encoded features, $f\left( {{P_n}} \right)$ and $f\left( {{M_n}} \right)$ for a prior patient are input into the DDL block. The goal of the DDL block is to subtract the initial segmentation and style segmentation in the feature space. DDL Block consists of a difference layer followed by a 3D convolution layer with tanh activation function. If multiple prior patients are available, the output of the tanh layer is averaged for all the prior features. The query data $\left\{ {{X_{{\text{new}}}},{ }{M_{{\text{new}}}}} \right\}$ is passed through the same encoder, and the resulting features are concatenated with the output of DDL block. The DDL Block can be succinctly defined by the equation ${\text{DDLBlock}}\left( {\left\{ {{P_1}, \ldots ,{P_n}} \right\},\left\{ {{M_1}, \ldots ,{M_n}} \right\}} \right)$ $= \frac{1}{n}\mathop \sum \limits_{i = 1}^n {\text{tanh}}\left( {{\text{Conv}}3{D_\theta }\left( {f\left( {{P_i}} \right) - f\left( {{M_i}} \right)} \right)} \right)$, where the learnable variables, $\theta $, parameterize the Conv3D operation. These features are then decoded back using a multi-layer CNN to produce the stylized segmentation. Each resolution level containing the same number of features that was previously mentioned (128, 64, and 32). The encoder, DDL Block, and decoder were trained simultaneously with a combined dice similarity coefficients (DSC) and Hausdorff distance (HD) loss [41, 42], as outlined in the training details section. This same architecture can be universally applied to any type of segmentation model and style adaptations.

### 2.3. Training details

A combination of DSC loss and HD-based loss was used for model training. DSC is a metric that measures the overlap between the predicted segmentation and the ground truth. It focuses on the pixel-wise overlap, making it sensitive to the overall shape and size of the segmented regions. HD measures the maximum distance between the boundary points of the predicted segmentation and the ground truth and is useful for capturing boundary accuracy. Both of these are relevant metrics that geometrically reflect what the clinicians are striving towards. This is important in medical imaging where precise boundary delineation is crucial for diagnosis and treatment planning. We acknowledge that these metrics may not necessarily reflect the accuracy of the segmentation with respect to dose discrepancies, as a high DSC error in a low dose gradient area may still be acceptable. We plan to investigate dosimetric impacts of segmentation error in a future study. We used an epoch varying weight to balance these two losses such that initially DSC loss has the larger value. Once DSC goes above 70%, the weighted HD-based loss has values similar to the DSC loss for any training sample. We used Adam optimizer with a learning rate of 10^−3^. The batch size was set to one. The model is trained to accept up to 10 prior patients.

The training data pipeline is as such:

1) A new CT image that we wish to segment is presented (query).
2) Multiple CTs with approved segmentations (${P_i}\forall i \in \left( {1,{\text{ rand}}\left( {10} \right)} \right)$) of an existing style are gathered (support). For training purposes, a random set of CTs, from 1–10 CTs, are used at each training iteration.
3) A generic segmentation model (Seg_model_) is used to segment the desired structure on the query CT and the support CTs. At this point in the pipeline, there is 1 segmentation from the query CT (${M_{{\text{new}}}})$, and 2 segmentations from each of the support CTs (${M_i},{ }{P_i}\forall i \in \left( {1,{\text{ rand}}\left( {10} \right)} \right)$).
4) All segmentations are processed into latent space data using the encoder, $f\left( \cdot \right)$.
5) The latent space representation of the segmentations from the support CTs, ($f({M_i}),{ }f\left( {{P_i}} \right)\forall i \in \left( {1,{\text{ rand}}\left( {10} \right)} \right)$) are processed through the DDL block defined by the equation ${\text{DDLBlock}}\left( {\left\{ {{P_1}, \ldots ,{P_n}} \right\},\left\{ {{M_1}, \ldots ,{M_n}} \right\}} \right) = \frac{1}{n}\mathop \sum \limits_{i = 1}^n {\text{tanh}}\left( {{\text{Conv}}3{D_\theta }\left( {f\left( {{P_i}} \right) - f\left( {{M_i}} \right)} \right)} \right).$
6) The output of the DDL Block, as well as the final and intermediate outputs from the encoder for the query CT, are fed into the decoder, which processes the information to produce the final segmentation, ${P_{{\text{new}}}}$.
7) ${P_{{\text{new}}}}$ is compared to the ground truth segmentation, ${P_{{\text{groundtruth}}}}$, as part of the loss function. The encoder, DDL Block, and decoder are all updated during the backpropagation step.

The model was trained to accept any number of prior patients from one to ten. At every iteration, the model is exposed to a random number of prior datasets. The Prior-guided DDL model is trained and designed with the assumption that data for the new style is unavailable at the time of training. The meta-learner should be capable of learning the difference in style for any new style encountered. To facilitate this, the meta-learner needs to be trained with multiple systematic style variations compared to the original structure. For meta-learner training, new styles were simulated from the available whole structure training data. The augmentations used for different style label simulation are detailed in the **Data Augmentation & Simulation** section.

For the segmentation model that produces prior segmentation, a 3D multi-task network was used for CTV segmentation [23], a 3D UNet++ was used for parotid segmentation [43], and a 3D UNet architecture was used for rectum segmentation [17].

### 2.4. Dataset

#### 2.4.1. Training data

Post-operative CTV dataset consists of 100 patients treated by a single clinician with adjuvant/salvage radiotherapy at UT Southwestern Medical Center (UTSW) from 2017 to 2020. At the UTSW genitourinary radiation oncology service, these clinical contours are usually drawn by residents, corrected by the supervising attending clinician, and reviewed by all attending clinicians. Each CT volume contains 60–360 slices and a voxel size of 1.17 × 1.17 × 2 mm^3^.

As for the parotid gland training dataset, we used the 120 open-access H&N CT scans of patients with nasopharyngeal cancer from the Automatic Structure Segmentation for Radiotherapy Planning Challenge [44]. Each CT scan was marked by one experienced oncologist and verified by another experienced oncologist. All of the original CT scans in this dataset consisted of 100–144 slices of 512 × 512 pixels, with a voxel resolution of ([0.98–1.18] × [0.98–1.18] × 3.00 mm^3^).

The whole rectum dataset consisted of 220 patients with prostate cancer treated at UTSW from 2017 to 2019, with raw CT scan images of 136 prostate cancer patients. All CT images were acquired using a 16-slice CT scanner (Royal Philips Electronics, Eindhoven, The Netherlands). Rectum was contoured by experienced radiation oncologists. All images were acquired with a 512 × 512 matrix and 2 mm slice thickness (voxel size 1.17 × 1.17 × 2 mm^3^).

#### 2.4.2. Testing data

For post-operative CTV segmentation, 30 patients were used for testing, with 3 style variations based on common variations observed across institutions and different guidelines [45–50]: (1) CTV_style1_ extending only 1 cm into Bladder; (2) CTV_style2_ with superior rectum included; and (3) CTV_style3_ excluding the seminal vesicles. Each CT volume contains 60–360 slices and a voxel size of 1.17 mm × 1.17 mm × 2 mm.

For rectum segmentation, two sub-structures were evaluated with Prior-guided DDL: Rectum_superior_ and Rectum_posterior_, representing the superior and posterior aspects of the rectum, respectively. 11 patients with Rectum_superior_ and Rectum_posterior_ contoured by expert clinicians were used for testing. All images were acquired with a 512 × 512 matrix and 2 mm slice thickness (voxel size 1.17 × 1.17 × 2 mm^3^).

For parotid gland segmentation, Parotid_superficial_ is used as the label variation for testing. Superficial parotid gland is just the superficial lobe of the parotid gland without the deep lobe included. 25 patients with superficial parotid contoured by an expert clinician at UTSW were used as the test dataset. All the scans in this dataset contain 124–203 slices of 512 × 512 pixels, with a voxel resolution of ([1.17–1.37] × [1.17–1.37] × 3.00 mm^3^).

The data that come from UTSW were natively extracted from clinical systems in DICOM format. These required additional preprocessing in order to convert to python-readable arrays. The DICOM data are first extracted into Python using the Pydicom package. DICOM structure files are point cloud data that define the edge of the contour. These can be converted to a 3D Boolean mask by the RT-Utils Python package. Using the Numpy package in python, we then convert these arrays to Numpy arrays and save the files for use in the project.

### 2.5. Data augmentation & simulation

For training the DDL meta-learner, multiple data augmentations were performed on the whole structure. For CTV dataset, the following data augmentations were performed: structure dilation, structure erosion, CTV completely excluding bladder, CTV 3 cm into bladder, CTV 2 cm into rectum, CTV contours only in slices with rectum and bladder. The structure dilation and structure erosion operations had dilated/eroded the contour edge by 2 pixels isometrically in all directions.

For the parotid gland, the data-augmentations used for training the meta-learner included structure dilation, structure erosion, parotid contours only on slices with masseter muscles, parotid contours only on slices with the spinal canal, and parotid contours only in slices without masseter muscles or the spinal canal.

For rectum dataset, the following data augmentations were performed: structure dilation, structure erosion, rectum contours only on slices with the bladder, rectum contours only in slices with the prostate, rectum contours only in slices without the prostate or bladder. These augmentations were chosen so that they are systematic but diverse enough to capture different kinds of variations possible.

### 2.6. Evaluation

To evaluate our model’s performance, we tested its ability to learn unseen structure styles. For the parotid and rectum, we used existing style variations: Rectum_superior_, Rectum_posterior,_ and Parotid_superficial_. For the CTV, we simulated three new style variations that are commonly observed across different institutions and guidelines.

## 3. Results

Table 1(a) presents the performance of the pre-trained general segmentation model on the whole rectum, parotid gland, and the existing CTV style. When pre-trained segmentation is compared against its own existing style, the DSC on test data set exceeds 85% for all three structures. However, when the general model was applied to the new styles, as shown in table 1(b), its performance dropped substantially, with the DSC falling as low as 46% for the superior rectum.

**Table 1.** Performance of the pre-trained general segmentation model when tested on the whole rectum, parotid gland, and existing CTV style (a) and when tested on the new styles (b).

(a)	
STRUCTURE	DSC (%) [Test dataset]
CTV	85.8 ± 6.80
Parotid gland	87.6 ± 3.40
Rectum	85.4 ± 4.70

(b)	
STRUCTURE	DSC (%) [Test dataset]
CTV_style1_	78.6 ± 6.29
CTV_style2_	71.9 ± 7.57
CTV_style3_	63.0 ± 9.56
Superficial parotid gland	69.6 ± 12.1
Rectum posterior	52.2 ± 12.4
Rectum superior	46.3 ± 7.60

**Figure 3.**
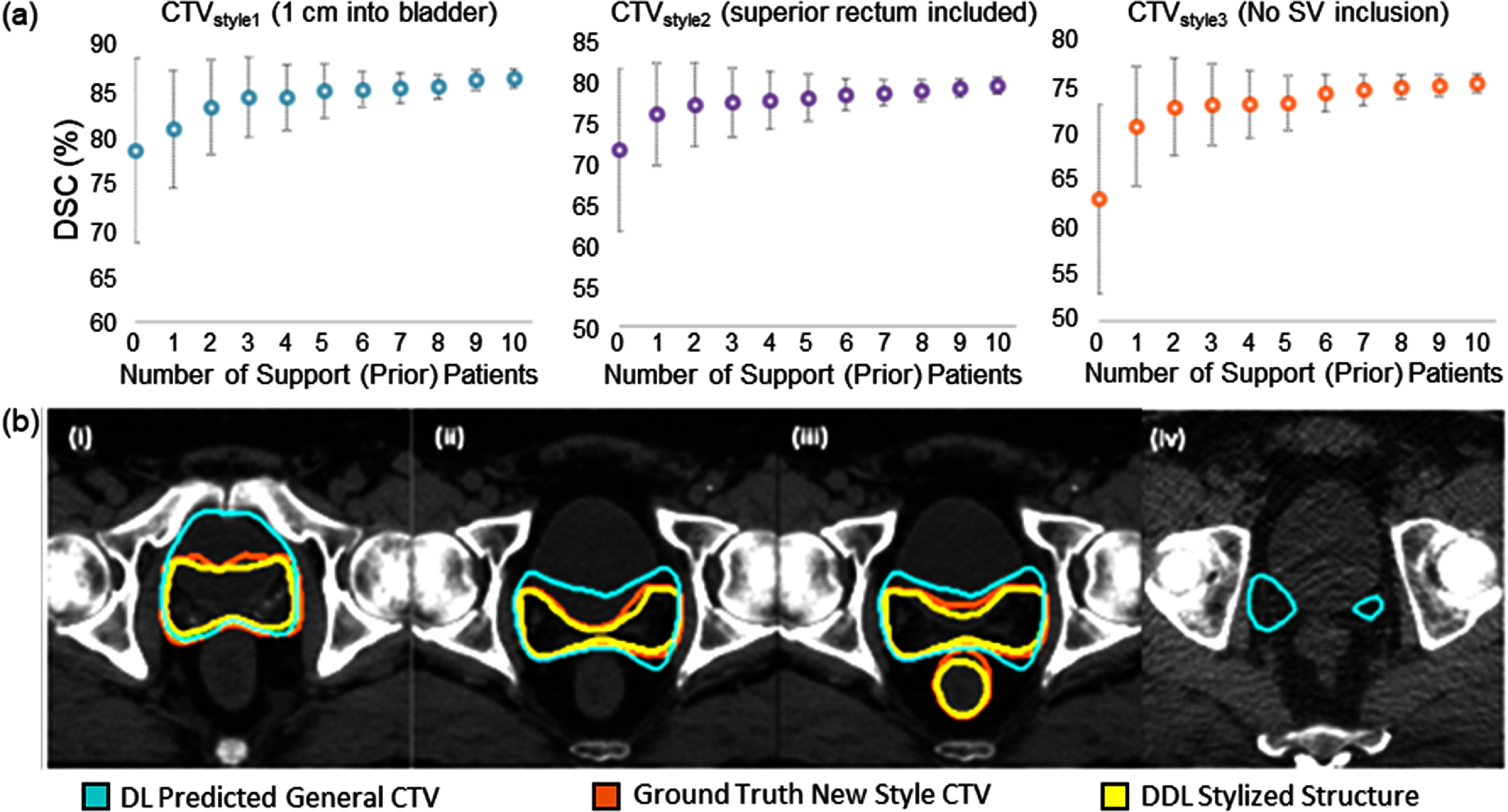
Showcase for the segmentation of CTV style variations. (a) The average DSC on the test dataset for Prior-guided DDL model. The plot shows the improvement in DSC as the number of input prior patients increases. (b) Visualization of DDL performance for CTV style variations with 5 prior patients as input (green: initial segmentation model prediction, red: ground truth CTV simulated in a new style, yellow: Prior-guided DDL stylized contour with 5 input prior patients. (i) shows an example for the CTV 1 cm into bladder, (ii) shows an example for the CTV completely excluding bladder, (iii) shows an example for CTV with superior rectum included and (iv) shows an example for the CTV with seminal vesicles excluded.

The performance of the Prior-guided DDL model on CTV style variations is shown in figure 3. Figure 3(a) presents a plot for the average DSC on the test dataset for the proposed model, highlighting the improvement in DSC as the number of input prior patients increases. Figure 3(b) shows a visualization of model performance for the simulated CTV styles with 5 prior patients as input. Figure 3(b)**(i)** shows an example for CTV 1 cm into Bladder, figure 3(b)**(ii)** shows an example for CTV completely excluding Bladder, figure 3(b)**(iii)** shows an example for CTV with superior rectum included and figure 3(b)**(iv)** shows an example for CTV with seminal vesicles excluded for a test patient.

**Figure 4.**
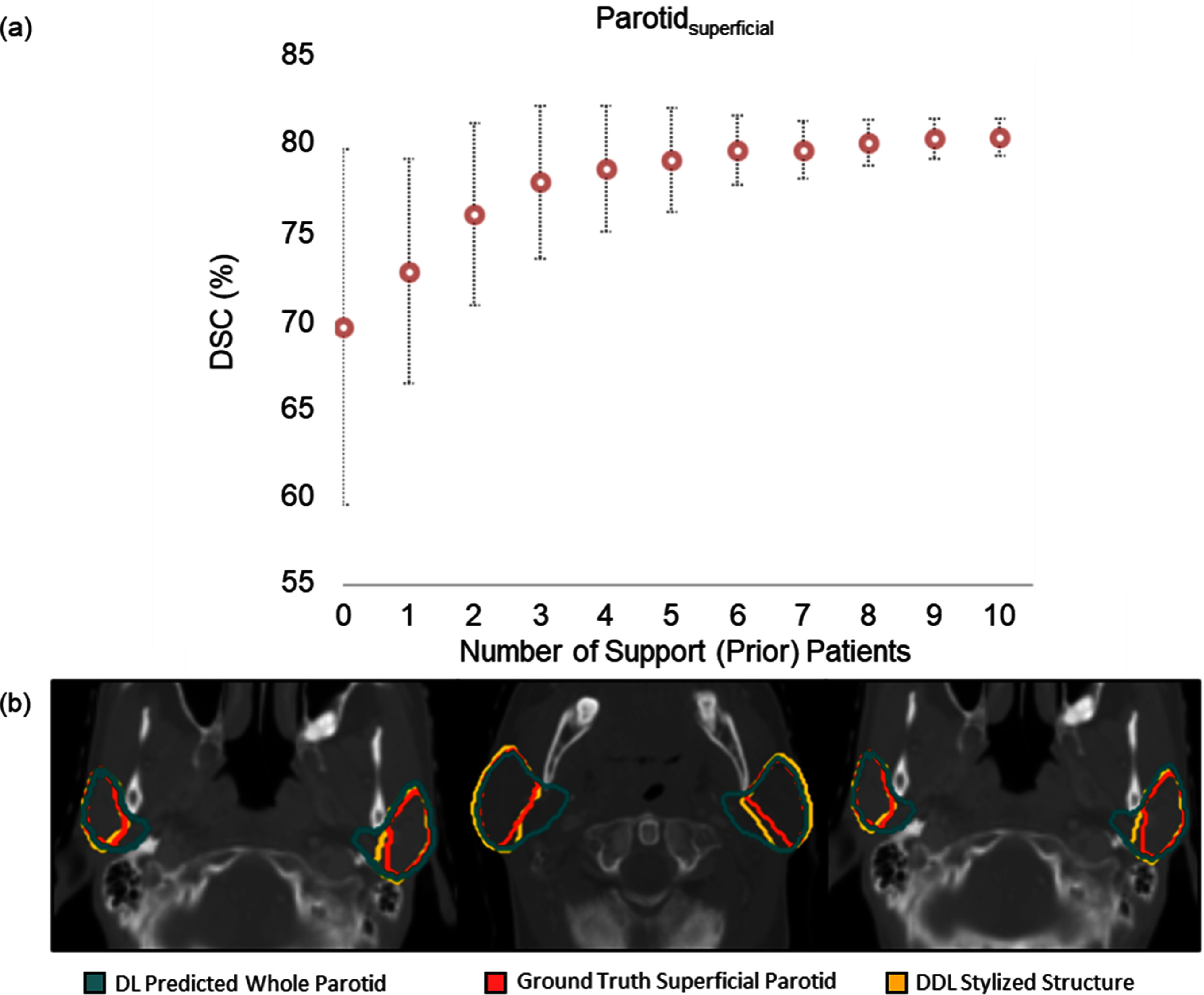
Showcase for the segmentation of Parotid_superficial_ gland. (a) The average DSC on the test dataset for DDL model. The plot shows the improvement in DSC as the number of input prior patients increases. The DSC improvement slows down after 6 prior patients. (b) Visualization of DDL performance for Parotid_superficial_ gland with 4 prior patients as input (green: whole parotid segmentation model prediction, red: ground truth superficial parotid gland contoured by clinician, yellow: DDL stylized contour with 4 input prior patients).

Figure 4 illustrates the performance of the Prior-guided DDL model on the Parotid_superficial_. Figure 4(a) shows the average DSC on the test dataset with the proposed model, comparing the performance against a varying number of support patients along the *x*-axis. As the number of input prior patients increases, the plot shows an asymptotic improvement in DSC. The DSC improvement slows down after 6 prior patients. Figure 4(b) provides a visualization of Prior-guided DDL performance for Parotid_superficial_ gland with 4 prior patients as input. With just 4 prior patients as input, it can be observed that the model is able to segment the Parotid_superficial_ more precisely compared to the whole structure segmentation model.

**Figure 5.**
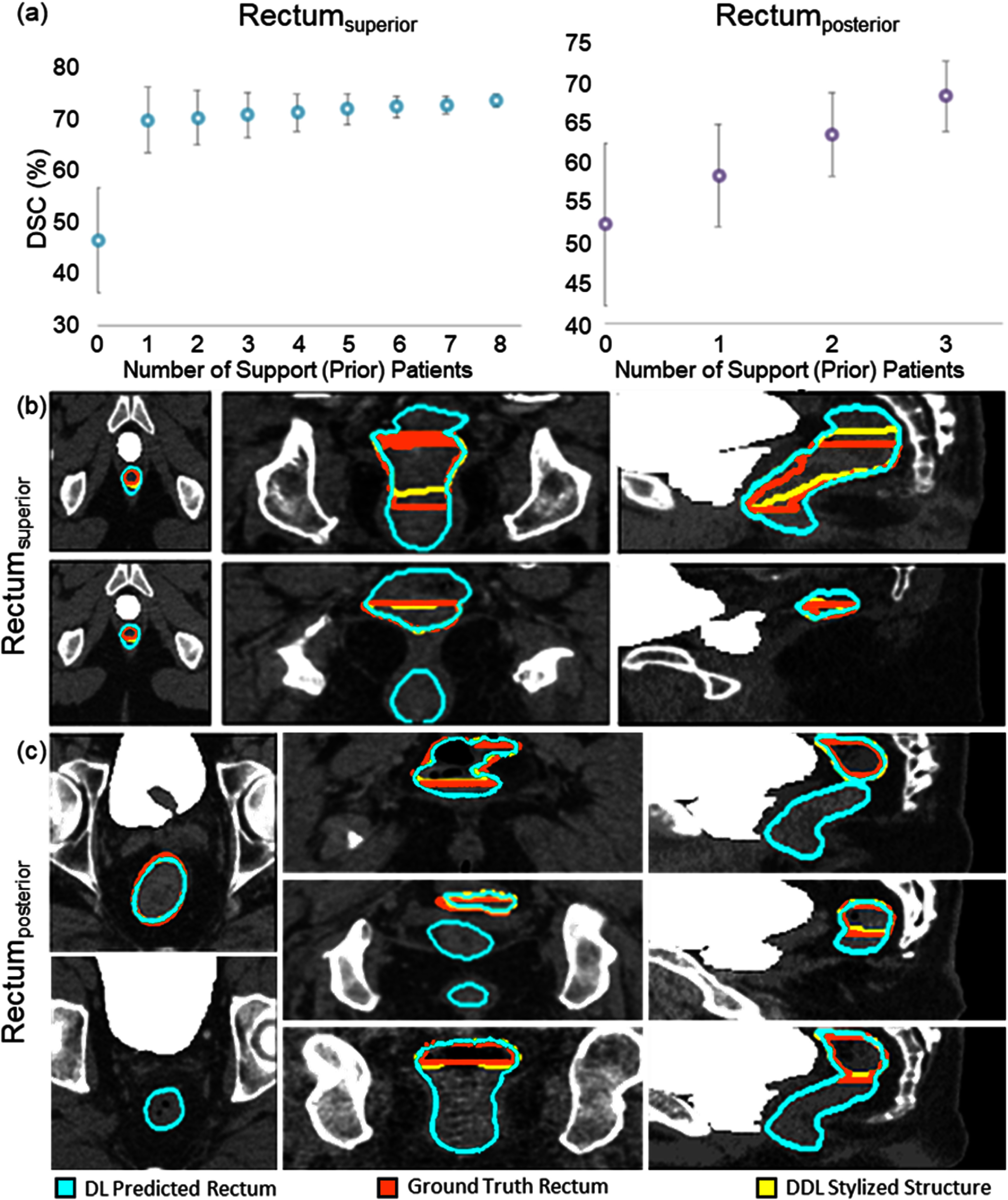
Showcase for the segmentation of posterior and Rectum_superior_. (a) The average DSC on the test dataset for DDL model. The plot shows the improvement in DSC as the number of input prior patients increases. (b) Visualization of model performance for Rectum_superior_ on axial, coronal and sagittal slices with 3 prior patients as input (green: whole rectum segmentation model prediction, red: ground truth Rectum_superior_ contoured by clinician, yellow: DDL stylized contour with 3 input prior patients. (c) Visualization of model performance for Rectum_posterior_ on axial, coronal and sagittal slices with 3 prior patients as input (green: whole rectum segmentation model prediction, red: ground truth Rectum_posterior_ contoured by clinician, yellow: DDL stylized contour with 3 input prior patients).

**Figure 6.**
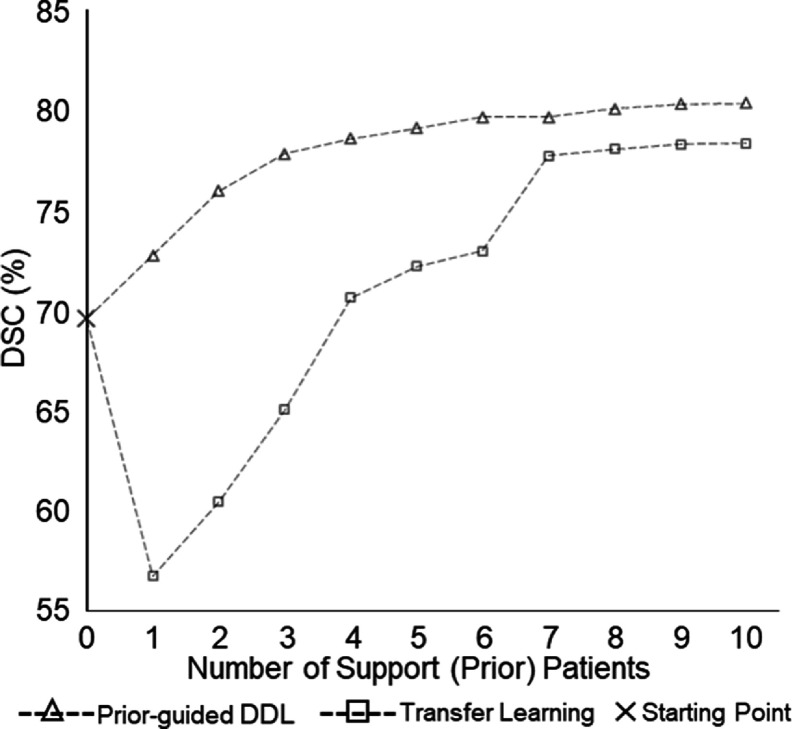
Comparison of the prior guided DDL model with transfer learning for model adaptation to Parotid_superficial_. The *x*-axis represents the priors used in prior-guided DDL model and the number of patients used for tuning the transfer learning model. It can be observed that since the number of patients is low, the transfer learning model does not perform as well as the prior-guided DDL model.

The performance of the Prior-guided DDL model on rectum labeling variations is visualized in figure 5. Figure 5(a) plots the average DSC on the test dataset. The DSC increases as the number of input prior patients increases. For Rectum_superior_ only three prior patients were available for testing. Figure 5(b) shows a visualization of model performance for Rectum_superior_ with 3 prior patients as input. Figure 5(c) shows a visualization of model performance for Rectum_posterior_ with 3 prior patients as input. For both Rectum_superior_ and Rectum_posterior_, it can be observed that the DDL stylized contour predicted a much more precise segmentation compared to the whole rectum segmentation model. For figures 3–6, the ‘no adaptation’ baseline corresponds to the model having 0 prior support patients.

To demonstrate the effectiveness the prior guided DDL model with just a handful of patients for improved segmentation, we compare the model with transfer learning. Figure 6 plots the performance of prior guided DDL model against transfer learning for Parotid_superficial_. *X*-axis represents the number of priors used in the prior-guided DDL model and the number of patients used for tuning the transfer learning model. It can be observed that since the number of patients is low, transfer learning model does not perform as well as prior-guided DDL model. In addition to comparing against the transfer learning baseline, we also compare the models to a ‘no adaptation baseline’ which represents the model’s performance with 0 prior support patients.

## 4. Discussion

We have proposed a new model capable of adapting to systematic differences in labeling styles across structures with only a handful of patient labels. This model directly addresses the issue of labeling variations that many deep learning models encounter when deployed in clinical practice. In this work, we introduce a prior-guided DDL that learns how to adapt the segmentation model’s prediction to a new desired style. Since the model is meta-trained, it can predict the new style from prior patients without updating any model parameters. This distinguishes it from other adaptation methods that require retraining or fine-tuning. The model only needs prior patient data of a particular target style to guide the new segmentation style and can use a flexible number of prior patient data to better average the style in question. In summary, we have shown that our model effectively adapts to new labeling styles—with improved DSC from 78.6, 71.9, 63.0, 69.6, 52.2, and 46.3–84.4, 77.8, 73.0, 77.8, 70.5, 68.1, for CTVstyle1, CTVstyle2, CTVstyle3, Parotid_superficial_, Rectum_superior_, and Rectum_posterior_, respectively—demonstrating its potential to improve segmentation accuracy and consistency in clinical practice. With just 3 prior patients, the prior-guided DDL model has drastically improved DSC values by 7%–20%. The major potential impact is that this framework will allow for fast and robust deployment of the segmentation model across different institutions with different styles. Without any additional training, the new institution only needs to provide a handful of patient samples with the segmentation style that they desire.

Our proposed method provided additional benefits over other existing methods. A recent study that looked at style adaptation for the auto-delineation of rectal cancer CTVs using attention mechanisms found promising results for adapting to different styles [51]. Their method entailed first training a baseline model using 172 cases and then adapting and retraining this baseline model on specific datasets from 2 different clinicians (79 and 64 training patients), to make 2 specific style models. The limitation of this method is that it requires training with a large number of patients, and the inflexibility of the trained models to adapt to newer styles outside of its training data. Another previous study that developed style-aware segmentation using PSA-Net, successfully identified and performed style transfer using transfer learning technique [52]. Across 4 different clinician styles, over 300 patients were used to train and validate the models that were trained. Unlike these 2 aforementioned methods, our proposed method is able to adapt to styles with (1) no additional training after the initial training, and (2) substantially fewer example patients—even as few as one patient, (3) not limited to the styles of segmentation of its original training data. Another relevant study looked at unsupervised domain adaptation through style adaptation and boundary enhancement for medical semantic segmentation [53]. This study was more focused on unsupervised segmentation across multiple image modalities and relying on boundary information. While this may work well for structures that have clear delineation, the method may struggle with contours that do not have defined boundaries, such as the CTV with cancerous invasion that is invisible in the image. Our proposed method is able to effectively handle CTV contouring due to the prior patient style data that is provided.

However, this study carries several limitations. The model proposed is capable of learning systematic style differences, but it will not be able to easily adapt when the differences are random. For example, a clinician who is given the same image to contour at 2 different times will not contour the image identically, which can be due to several factors such as their fatigue and judgment that day, clinical time constraints and stresses, different environmental conditions, interruptions, and their emotional state that day. These random differences cannot be captured by current modeling methods, and the resulting segmentation will include an average of these errors. In addition, if there is high variability or noise in the data itself (e.g. poor CT image quality with a lot of artifacts), this may also reduce the model’s segmentation accuracy. Additionally, this approach leaves the selection of patient style and data entirely up to the user. Poor or conflicting selection of prior patient data may degrade the final segmentation performance. We also did not explore the impact on performance once the style is outside of the ranges of the styles that the model trained on. Currently, the study uses a variation of styles that are clinically relevant, but does not push the boundaries of performance if a unique edge case presents specific anatomical variations.

We also have not exhaustively explored other training parameters to further improve performance, such as investigating additional losses to use in combination with our DSC and/or HD losses. In addition, the number of patients used for testing is relatively small. While it is adequate for a proof-of-concept study, including larger and more diverse datasets would provide a much better conclusion and indicate the model’s ability to generalize to other clinical styles. It is also unclear to what extent of the truly meaningful impact of our proposed framework will be with a better DSC or HD. While there is a correlation between segmentation similarity metrics and the contour editing time, the exact impact for postoperative prostate cancer patients and other cancer sites in radiation oncology is unknown until dosimetric and clinical evaluations take place. We plan to expand on this study by clinically implementing the usage of the model in a more realistic setting and investigating dosimetric impacts. We will expand the data size and evaluate such types of edge cases in a more rigorous reader-type study, as clinicians can then assess the clinical impact of such errors and provide meaning to the subtle differences we measure in DSC.

Deploying deep learning models in clinical settings presents challenges in hardware, workflows, and real-time performance. Although the GPU required for deployment is smaller than the one used for training, a GPU-equipped clinical server remains necessary. This is manageable for larger centers, but it can be problematic for smaller community centers. Integrating the model into clinical workflows can be done in two ways: (1) as standalone software or (2) via API integration with existing clinical software. Standalone software is more portable, but often requires manual data transfers, increasing the clinician’s workload. API integration offers a smoother experience, but demands skilled developers familiar with clinical systems, a resource that many clinics lack. While most deep learning models can infer in fractions of a second, performance bottlenecks often occur during data export/import and processing, which can take several minutes. Addressing these data transfer issues is crucial for achieving real-time performance.

## 5. Conclusion

The study demonstrates that the Prior-guided DDL effectively adapts pre-trained auto-segmentation models to new clinical styles with minimal effort. By learning the systematic differences between the general model predictions and clinician-approved contours from a small initial patient group, the Prior-guided DDL substantially improves segmentation accuracy across various anatomical structures. This approach offers a practical solution for institutions to customize segmentation models to meet their specific needs without extensive retraining, enhancing the precision and efficiency of radiotherapy treatment planning.

## Data Availability

The data cannot be made publicly available upon publication because they contain sensitive personal information. The data that support the findings of this study are available upon reasonable request from the authors.

## Data and code availability

The data and source code that support the findings of this study are available from the corresponding author upon reasonable request.
